# Isometric trunk strength in seated para-athletes: relationship to manual muscle testing and comparison with non-impaired controls

**DOI:** 10.3389/fspor.2026.1888235

**Published:** 2026-09-29

**Authors:** Marie Lund Ohlsson, Frida Lidén, Johanna S. Rosén, Kajetan Ciunelis, Karina Sá, Yves Vanlandewijck, Anna Bjerkefors

**Affiliations:** 1 Department of Physiology, Nutrition and Biomechanics, Swedish School of Sport and Health Sciences (GIH), Stockholm, Sweden; 2 Department of Health Sciences, Swedish Winter Sport Research Centre, Mid Sweden University, Östersund, Sweden; 3 PhD School, The Józef Piłsudski University of Physical Education in Warsaw, Warsaw, Poland; 4 Physical Education Graduate Program, Universidade Estadual de Campinas, Campinas, SP, Brazil; 5 Department of Neuroscience, Karolinska Institutet, Stockholm, Sweden

**Keywords:** classification, disability sports, muscle strength assessment, neuromusculoskeletal impairment, parasports

## Abstract

**Introduction:**

Trunk muscle strength is an important performance indicator in para-athletes competing in seated sports and is commonly assessed in classification systems using manual muscle testing (MMT), together with functional and sport-specific tests. Quantitative isometric strength measures may provide complementary information about force-generating capacity. However, the relationship between isometric trunk strength and MMT-assessed trunk and hip strength is not well described in para-athletes with neuromusculoskeletal impairments. This study aimed to examine isometric trunk strength in seated para-athletes and compare it with 1) MMT scores of the trunk and hips and 2) isometric trunk strength in a reference group without physical impairments.

**Methods:**

Male para-athletes competing in seated sports (*n* = 32, 34.7 ± 13.7 years, 8.7 ± 4.1 exercise h/week) and a reference group without physical impairments of similar age and weekly exercise (*n* = 10, 35.0 ± 12.5 years, 9.8 ± 6.4 exercise h/week) participated. Isometric strength was measured seated for flexion, extension, rotation, and lateral flexion using a strength rig. MMT of the trunk and hips was summed to obtain an MMT Trunk–Hip score. Associations with isometric strength were examined using Spearman's rank correlation. Para-athletes were categorized into Very Low, Low, Moderate, and High MMT Trunk–Hip groups. Group differences were analyzed using Kruskal–Wallis tests.

**Results:**

Higher MMT Trunk–Hip scores were associated with greater maximal isometric trunk strength in all movement directions (*p* < 0.001), with large effect sizes. The High group did not differ from the reference group, whereas the Very Low and Low groups consistently demonstrated lower strength across directions. The Moderate group showed greater heterogeneity, with strength varying by movement direction.

**Conclusion:**

MMT Trunk–Hip scores were strongly associated with isometric trunk strength in seated para-athletes, supporting MMT's ability to capture meaningful differences in trunk and hip muscle strength. However, direction-specific variation indicates that MMT does not fully characterize force-generating capacity. Quantitative isometric strength testing may provide complementary information for evaluating and monitoring trunk strength, particularly for performance enhancement. In para-sport classification, these findings support the construct validity of MMT while highlighting the potential value of quantitative strength measures for developing and validating multidimensional assessment methods.

## 1 Introduction

The trunk includes the spine, thorax, pelvis, and hips, along with the muscles that support the lumbopelvic-hip complex (1). The trunk is important for stabilizing everyday tasks (2) and for transferring forces between limbs during dynamic tasks such as kicking, throwing, and running (3). The trunk muscles provide proximal stability for distal mobility, implying that the trunk is an important link of the kinetic chain for arm and leg movement. This role is particularly important in sports performance, where effective force transfer is essential for upper-limb actions (4).

Para-athletes with neuromusculoskeletal impairment, affecting trunk and/or leg muscle function, represent a heterogeneous group, with varying types of impairment influencing movement capacity (5). Reduced trunk and leg function limits walking and running ability, and many para-athletes therefore compete in seated sports. In the Paralympic Games, many sports are available for athletes competing in a seated position, such as wheelchair court sports (basketball, rugby, tennis), para-athletics, and seated winter sports (para ice hockey, para cross-country skiing, and para-alpine skiing) (6).

For para-athletes in seated sports, the trunk and hip muscles play a central role for performance as the first link in the kinetic chain, transferring force and power from the seat to the arms (7). In the seated position, the degree of impairment in both trunk and hip muscle strength is relevant to sports performance. For example, less impaired trunk-hip muscle strength is associated with increased reach, greater upper limb force, and enhanced sports performance (e.g., faster wheelchair propulsion and longer or faster throws) (8–13). As trunk muscle strength directly influences performance and varies across impairment types and severity, it is also a key consideration in parasport classification.

The classification systems aim to ensure fairness by minimizing the impact of impairment on performance, determining eligibility to compete, and grouping athletes into sport classes where sporting excellence determines the winner (5). Therefore, an evidence-based classification system for seated parasport should have a valid assessment of strength impairment of the trunk and an established relation between the measures of strength impairment and the sport-specific measures of performance (14, 15). However, the methods used to assess muscle strength within classification systems remain debated (16, 17).

In many parasport classification systems, manual muscle testing (MMT) is used to assess impairment of muscle strength, often supporting the minimal impairment criteria (eligibility to compete) and also sometimes supporting sport class allocation. MMTs are simple clinical tests requiring no instrumentation and provide a judgment of the ability to activate a muscle group (low levels of muscle strength) on an ordinal scale based on the ability to move against gravity and resistance. However, MMT has important limitations for assessing muscle strength, including ceiling effects, reliance on qualitative judgments, and limited information about higher levels of muscle strength (17). Although MMT is not an objective measure, it provides a clinically feasible assessment of isolated muscle strength. When performed using standardized procedures, it has been shown to demonstrate acceptable validity and reliability (18–20).

Isometric strength has been proposed as a better method to assess muscle strength compared to MMT in populations with physical impairments due to its objectivity and resulting in ratio-scaled variables possible to associate with sport-specific performance test results (21). Another reason supporting isometric strength is the assumption of its low influence of fitness or training status in populations with physical impairment (21). However, the evidence supporting this assumption is limited, and in non-impaired populations, isometric strength is clearly trainable (1, 22). The paper by Beckman (21) did not, for example, include athletes with incomplete spinal cord injury (SCI) in their discussion, despite this subgroup representing a growing proportion of the SCI population due to advances in medical treatment (23). It has also been shown that maximal isometric strength increases over two years after a SCI, while the MMT scores do not increase after the acute phase (24). Indicating that MMT scores are not influenced by increases in strength over time, which isometric strength might be. This raises questions about both the validity of isometric strength as an assessment method of trunk impairment and the extent to which such measures translate to sport-specific performance and inform classification.

As trunk muscle strength is an important factor for performance in seated para-athletes, assessment methods are needed from both performance-enhancement and classification perspectives. From a performance perspective, such assessments can help monitor and support an individual athlete's fitness level and identify aspects of strength that may be trainable, while accounting for impairments that may limit voluntary muscle activation, such as paralysis. In classification, assessments should be resistant to training effects and are therefore typically based on multiple stages of evaluation. These may include MMT and functional tests, as well as sport-specific tasks and activities performed with the adaptive equipment used by the athlete and, when relevant, observations during competition (25). For example, in para-canoe classification, this is addressed by combining MMT-based assessments of trunk and hip strength with sport-specific testing performed in the athlete's adaptive equipment, including on-water evaluations (20, 26). MMT scores and functional test results have also been shown to be associated with performance and to demonstrate acceptable reliability (20).

Despite the relevance of trunk strength for seated para-athletes, it remains a poorly understood area in this population. Therefore, the aim of this study was to examine isometric trunk strength in seated para-athletes, and compare that to 1) their MMT scores of trunk and hips, and 2) isometric trunk strength of a reference group without physical impairments.

## 2 Material and methods

### 2.1 Study overview

This study included assessments of maximal isometric trunk strength in para-athletes competing in seated sports and in a reference group (Ref AB) without neuromusculoskeletal impairments. MMT of trunk and hips was also assessed in the para-athlete group. Data were collected in Sweden (para-athletes and Ref AB) and in Poland (para-athletes only).

Participants were recruited through various means, including online advertisements, social media (Facebook pages related to parasports and adapted physical activity), sports facilities, rehabilitation facilities, and sports clubs. Inclusion criteria for both groups were age 18–60 years. For para-athletes, additional criteria were: 1) training and competing in seated sports at national or international level, 2) having a health disorder resulting in a neuromusculoskeletal impairment (e.g., SCI, spina bifida, polio, leg amputation or dysmelia) affecting trunk and/or leg function (e.g., muscle strength, range of motion or limb deficiency), and 3) more than 1.5 years after acquired injury. For the Ref AB, additional criteria were: 1) engaging in exercise ≥3 days/week, and 2) no injuries or impairments affecting trunk and leg function. A formal *a priori* sample size calculation was not feasible because no directly comparable effect sizes were available for the association between isometric trunk strength and MMT scores of the trunk and hips. Sample size was therefore based on the available eligible population and informed by sample sizes reported in previous studies of strength in para-athletes with physical impairments (27–30).

A total of 32 para-athletes, wheelchair basketball (*n* = 16), wheelchair fencing (*n* = 7), para ice hockey (*n* = 3) and others (*n* = 6, weightlifting, wheelchair curling, table tennis, triathlon, rugby) and 10 participants in the Ref AB were included in the study (Table 1). All participants received oral and written information about the study before signing informed consent. The study was approved by the Swedish Ethical Review Authority (Dnr 2023-00990-01, and 2024-02137-01), and the Senate Commission of Research Studies Ethics of the Józef Piłsudski University of Physical Education in Warsaw (SKE 01-28/2024).

**Table 1:** Demographics and isometric trunk strength of para-athletes (*n* = 32) and the reference group without impairments (*n* = 10; Ref AB). Age, weight, height, weekly exercise amount, and maximal isometric strength (N/kg) in flexion, extension, rotation, and lateral flexion are presented as median (interquartile range) together with *p*-value (Mann Whitney U-test) and effect size (rank-biserial correlation). Significant differences (*p* < 0.05) marked in bold.

	Para-athletes	Ref AB	*p*-value	Effect size
N	32	10		
Age [years]	32.5 (22.8–43.0)	30.5 (26.3–44.3)	0.723	0.078
Weight [kg]	76.0 (67.4–86.4)	82.0 (79.0–85.7)	0.148	0.309
Height [m]	1.75 (1.69–1.80)	1.83 (1.77–1.87)	0.010	0.548
Exercise [hours/week]	9.0 (6.0–10.0)	6.8 (5.3–13.8)	0.893	−0.031
Maximal isometric strength				
Flexion [N/kg]	5.97 (2.90–7.44)	7.48 (7.43–7.98)	0.018	0.500
Extension [N/kg]	2.24 (1.25–5.89)	14.44 (14.13–15.18)	< 0.001	0.888
Rotation [N/kg]	4.07 (2.11–4.91)	6.51 (5.96–6.62)	0.003	0.625
Lateral flexion [N/kg]	2.65 (1.84–4.50)	6.49 (5.37–7.74)	< 0.001	0.738

### 2.2 Outcomes

Participants’ self-reported age, stature, weekly amount of exercise and health disorder (including the level of SCI). The participant's weight was measured on a scale with an accuracy of 0.1 kg, as the difference between the athlete + wheelchair and the wheelchair. Maximal isometric trunk strength tests were performed in a seated position for all participants in the following directions: flexion, extension, rotation (right and left), and lateral flexion (right and left) (Figure 1). The trunk and hips MMT in the para-athletes were assessed by two physiotherapists trained in the procedure.

**Figure 1:**
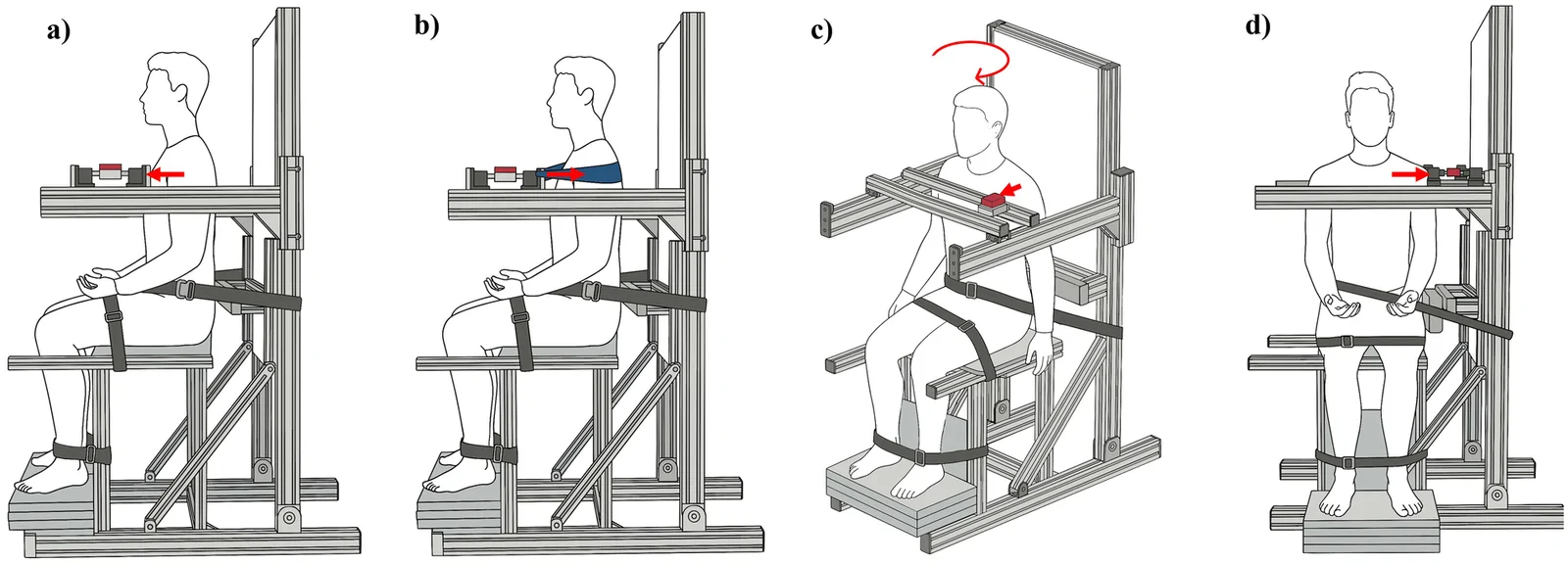
The isometric strength rig with strapping of pelvis and legs for **(a)** flexion, **(b)** extension, **(c)** rotation and **(d)** lateral flexion. The red box shows the position of the force transducer, and the arrows show the direction of the force (F) pulling or pushing on the transducer. Rotation and lateral flexion were performed in both left and right direction, the complementary direction of c and d are the mirrored figures.

Maximal isometric trunk strength was assessed in a seated position (90 degrees in hip and knee flexion) using a custom-made strength rig (Figure 1) with a height-adjustable sliding bar equipped with a force transducer (SLC-SMS-500 kg, Smart Load Cell, Sure Torque Europe, sampling frequency 100 Hz). Participants performed one familiarization trial (submaximal) followed by two maximal trials in each direction: trunk flexion, extension, rotation (right and left), and lateral flexion (right and left). A rest period of at least 30 s was provided between trials. During testing, participants were instructed to rest their palms on their laps and avoid using their arms to generate force. They were asked to gradually increase force over a 3-second period, followed by a maximal effort for 3 s. Participants were verbally encouraged by test leaders during the maximal effort phase during all trials. For flexion, extension, and rotation, participants were stabilized with straps over the pelvis and upper and lower legs, with additional support from a footrest and a pelvic backrest (Figures 1a–c). For lateral flexion, participants were repositioned to the side in the rig, with adjusted strapping, while maintaining fixation of the pelvis and legs; as a result, the pelvic backrest provided support to the lateral aspect of the pelvis (Figure 1d).

During flexion, rotation, and lateral flexion, participants pushed against the bar, whereas during extension, they pulled on the bar connected to the force transducer (Figures 1a–d). The height of the foot support was adjusted individually to 90 degrees in knee flexion. In flexion and extension, the force transducer was positioned at the midpoint of the sliding bar and approximately 2 cm below the top of the sternum. For rotation and lateral flexion, the force transducer was aligned with the shoulder (at the level of the glenohumeral joint) for each side, left and right. Participants exerted force on the bar in both directions (i.e., right and left rotation, and right and left lateral flexion). The maximal force (N) was recorded, and the highest value of two trials was used for further analysis. Because the aim of the study was not to study side differences (asymmetries), the mean of the left and right sides was reported for rotation and lateral flexion.

Trunk MMT was assessed using the protocol described in the Guidelines for the Trunk Test for Paracanoe Athletes (20, 26), with adjustments based on Daniels and Worthingham's Muscle Testing protocol (31). Tests were performed in the supine or prone position, depending on the movement assessed. The following trunk movements were tested: flexion, extension, rotation (right and left), lateral flexion (right and left), and lumbar/hip extension. The protocol uses a 0–2 scale, where 0 indicates no or minimal visible or palpable muscle contraction; 1 indicates partial range of motion with identifiable muscle contraction; and 2 indicates full range of motion against gravity.

The hip MMT assessment followed the Daniels and Worthingham's Muscle Testing (31). The following hip movements were tested bilaterally: flexion, extension, adduction, and abduction. The Oxford Scale ranges from 0 to 5, where 0 indicates no visible contraction, and 5 indicates full movement against gravity with maximal resistance.

To align with the trunk scoring system, hip MMT scores were downscaled to a 0–2 scale as follows: 0–1 was recoded as 0, 2–3 as 1, and 4–5 as 2. The maximum scores were 16 for the hips and 14 for the trunk. The sum of the scores, MMT Trunk and MMT Hip, was defined as MMT Trunk–Hip with maximum score 30.

### 2.3 Data analysis

Isometric strength was normalized to body weight. For rotation and lateral flexion, the mean of the left and right sides was reported. Normality of the isometric strength variables was assessed using Shapiro–Wilk tests, supported by visual inspection of histograms and Q–Q plots. These analyses indicated that most variables (≥ 75%) were not normally distributed. Accordingly, data were presented as medians and interquartile ranges (IQR), and non-parametric statistical methods were applied. Comparison between the Para-athlete and the Ref AB groups was performed using the Mann–Whitney U test; effect size was computed with rank-biserial correlation ($r_{\mathrm{rb}}=1-\frac{2U}{n_{1}n_{2}}$, U – Mann–Whitney test statistics, $n_{1}$ -sample size of group 1, $n_{2}$ -sample size of group 2)

Correlation analysis between isometric strength (normalized to body weight) and MMT Trunk-Hip was performed with Spearman's rank correlation (*ρ*) in all directions: flexion, extension, rotation and lateral flexion. To further examine the relation between isometric strength and MMT results, grouping strategies based on MMT results were explored. Cluster analysis was performed using the k-means method, specifying between one to eight clusters, based on Euclidian distance of the MMT Trunk-Hip scores in the para-athletes. Silhouette analysis did not identify an optimal number of clusters within this range; instead, the results indicated a linear distribution of MMT Trunk-Hip (Figure 2a). Given the absence of a distinct clustering structure, the para-athletes were therefore divided into four equally sized groups, the quartiles of the MMT Trunk-Hip scores: Very Low, Low, Moderate, and High function. Choosing four groups was based on the classification practices in wheelchair basketball, as this was the main population in the study, and comparable to the study of Schaaf (28). The Kruskal–Wallis test was used to assess differences in isometric strength between groups (Very Low, Low, Moderate, High). Effect sizes were calculated using epsilon squared ($\varepsilon^{2}=\frac{H-k+1}{N-k}$, H – Kruskal–Wallis test statistic, k – number of groups, N – total sample size). *Post hoc* pairwise comparisons between the groups (Very Low, Low, Moderate, High) were performed using the Mann–Whitney U test with Bonferroni correction, and effect size was computed with $r_{\mathrm{rb}}$. The same methods were used for comparison between the High and the Ref AB groups.

**Figure 2:**
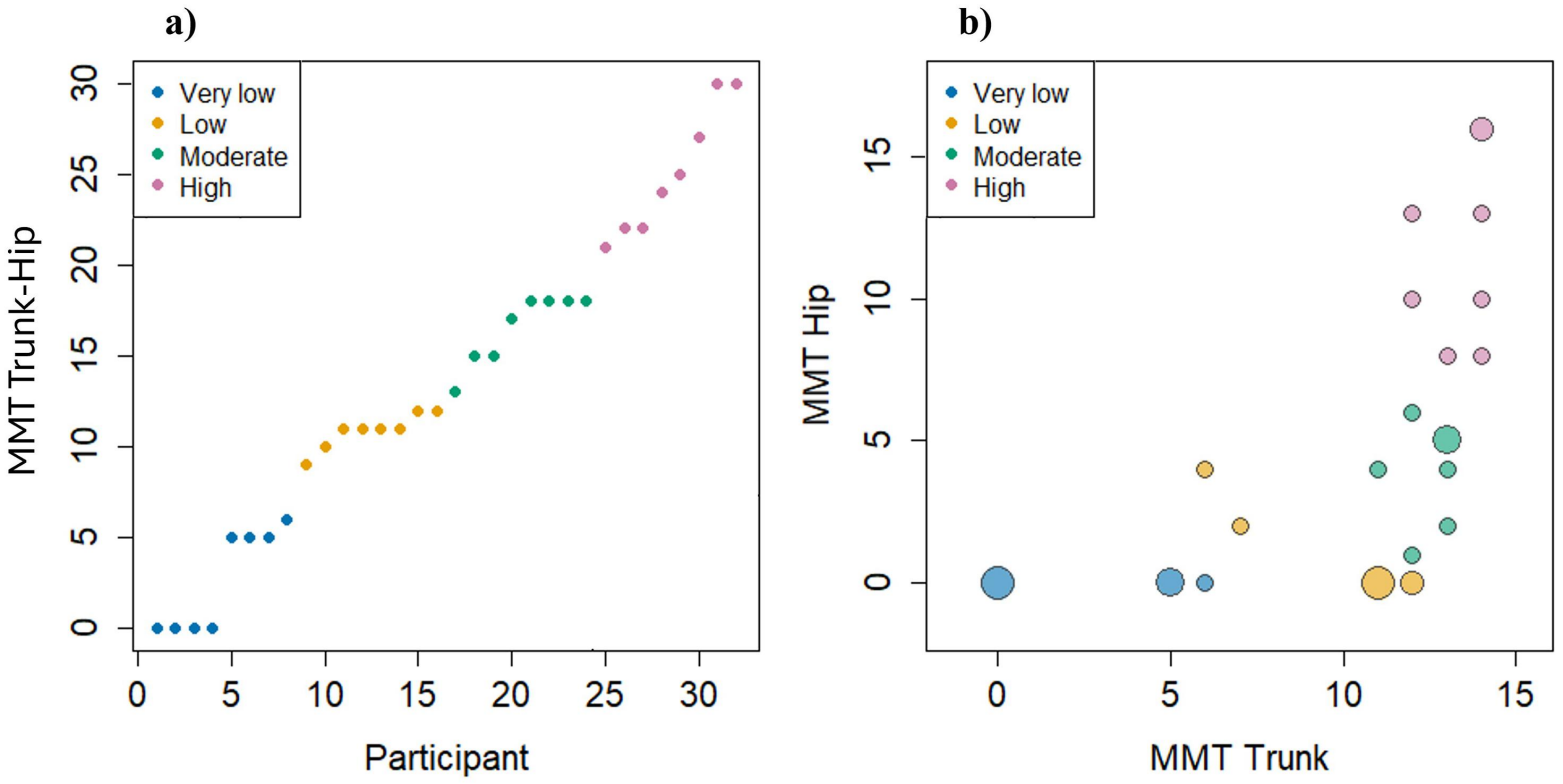
Distribution of MMT Trunk and Hip scores in para-athletes (*n* = 32) grouped based on MMT Trunk-Hip scores: Very low (blue), Low (yellow), Moderate (green), and High (pink): **(a)** total MMT Trunk and Hip score for all participants and **(b)** distribution of MMT Trunk and Hip scores. In **(b)**, the circle size is proportional to the number of participants at each score.

Statistical analyses were performed using R (RStudio 2024.12.1 build 563, Posit Software PBC). Statistical significance was set at *p* < 0.05. Effect sizes of $\varepsilon^{2}\;$ were interpreted according to Cohen's guidelines for ANOVA, with definitions for small (0.01 ≤  $\varepsilon^{2}$ < 0.06), medium (0.06 ≤  $\varepsilon^{2}$ < 0.14), and large ($\varepsilon^{2}$≥ 0.14). The effect sizes of $r_{\mathrm{rb}}$ were interpreted according to Cohen's guidelines for effect sizes as small (0.1 ≤  $r_{\mathrm{rb}}$  < 0.3), medium (0.3 ≤  $r_{\mathrm{rb}}$  < 0.5), and large (0.5 ≤  $r_{\mathrm{rb}}$) (32).

## 3 Results

Participant characteristics are presented in Table 1, showing no difference between para-athletes and the Ref AB group in age, weight, and amount of weekly exercise, while height was different between the groups. Isometric strength was higher in the Ref AB group, with large effect size in all directions.

The interquartile range of isometric strength in the para-athletes indicated large differences within the group (Table 1). Correlation analysis within the para-athlete group showed that isometric strength was associated with MMT Trunk-Hip scores in all directions: flexion (*ρ* = 0.78, *p* < 0.001), extension (*ρ* = 0.83, *p* < 0.001), rotation (*ρ* = 0.83, *p* < 0.001), and lateral flexion (*ρ* = 0.78, *p* < 0.001). Showing that higher MMT Trunk-Hip was associated with higher isometric strength.

The variation of isometric strength within the para-athletes group was explored based on MMT Trunk-Hip. As cluster analysis did not show an optimal number of clusters, para-athletes were manually grouped into four equally sized groups (*n* = 8 in each group) based on their MMT Trunk-Hip scores (sum of MMT Trunk and MMT Hip). The distribution of trunk and hip MMT scores in para-athletes is shown in Figure 2. The groups showed the following MMT Trunk-Hip scores: Very Low 0–6 points, Low 9–12 points, Moderate 13–18 points, and High 21–30 points. Remark: No participant had a score of 7, 8, 19 or 20. Generally, participants had lower MMT Hip than MMT Trunk scores (Figure 2b).

The characteristics of the sub-groups of para-athletes and their distribution of MMT scores and isometric strength are shown in Table 2. Isometric strength differed significantly between groups (Very Low, Low, Moderate, and High) in all directions. The overall pattern showed that groups with higher MMT Trunk-Hip scores had higher isometric strength. The distribution of isometric trunk strength across groups and significant differences between groups are presented in Figure 3. *Post hoc* analyses revealed no significant differences in any direction between the Very Low and Low groups. Flexion strength tended to be higher than extension for the Very Low and Low groups, while in the High group extension was clearly higher than flexion strength. Para-athletes with moderate MMT scores displayed a more heterogeneous, direction-specific pattern compared to the other groups. All significant differences of *post-hoc* tests had large effect sizes. Comparison between the isometric strength of the High group and the Ref AB group showed no difference in any direction.

**Table 2:** Demographics and isometric trunk strength of para-athletes (*n* = 32) grouped according to MMT Trunk-Hip scores into Very low (0–6 points), Low (9–12 points), Moderate (13–18 points), and High (21–30 points). Age, weight, height, weekly exercise amount, and maximal isometric strength in flexion, extension, rotation, and lateral flexion are presented as median (interquartile range) together with H-value (Kruskal–Wallis) with associated *p*-value and effect size (*ε*^2^). Significant differences (*p* < 0.05) marked in bold.

	Very low	Low	Moderate	High	H-value	*p*-value	Effect size
N	8	8	8	8			
MMT Trunk-Hip	0–6	9–12	13–18	21–30			
MMT Trunk	0–6	9–12	11–13	12–14			
MMT Hip	0	0–4	1–5	8–16			
Medical diagnosis	5 SCI (Th3-Th12), 3 SB	5 SCI (Th8–12), 3 SB	4 SCI (Th11-L4), 4 SB	3 AMP, 3 SCI (Th11-L4), 2 SB			
Age [years]	31.5 (21.8–42)	37 (28.8–45.2)	25 (22.5–29.8)	37.5 (30.5–50.2)	2.99	0.39	< 0.001
Weight [kg]	79.0 (67–83.1)	67.5 (60.1–81)	80.1 (73.9–90)	71.0 (67.2–87.2)	2.51	0.47	−0.018
Height [m]	1.68 (1.67–1.79)	1.74 (1.71–1.79)	1.75 (1.71–1.76)	1.78 (1.69–1.85)	1.38	0.71	−0.060
Exercise [hours/week]	9.5 (6–10.1)	7 (5.8–8.5)	10 (9.2–13.5)	8.5 (6.2–10)	3.44	0.33	0.016
Maximal isometric strength							
Flexion [N/kg]	2.5 (1–3.2)	3.9 (2.7–4.9)	6.6 (6–7.9)	7.9 (7.2–9.1)	18.51	< 0.001	0.554
Extension [N/kg]	1 (0.4–1.8)	1.2 (0.9–1.7)	4 (2.3–5.4)	10.7 (8.2–14.5)	22.65	< 0.001	0.702
Rotation [N/kg]	1.8 (0.6–2.3)	2.3 (2–3.8)	4.5 (4.1–5.1)	6.7 (5.2–7.2)	19.89	< 0.001	0.603
Lateral flexion [N/kg]	1.8 (0.9–2.5)	1.8 (1.6–2.1)	3.9 (3.2–4.7)	6.6 (4.4–7.1)	21.70	< 0.001	0.668

MMT, manual muscle testing; SCI, spinal cord injury, Th, thoracic level of SCI; L, lumbar level of SCI; AMP, amputation of lower leg/s, SB; spina bifida.

**Figure 3:**
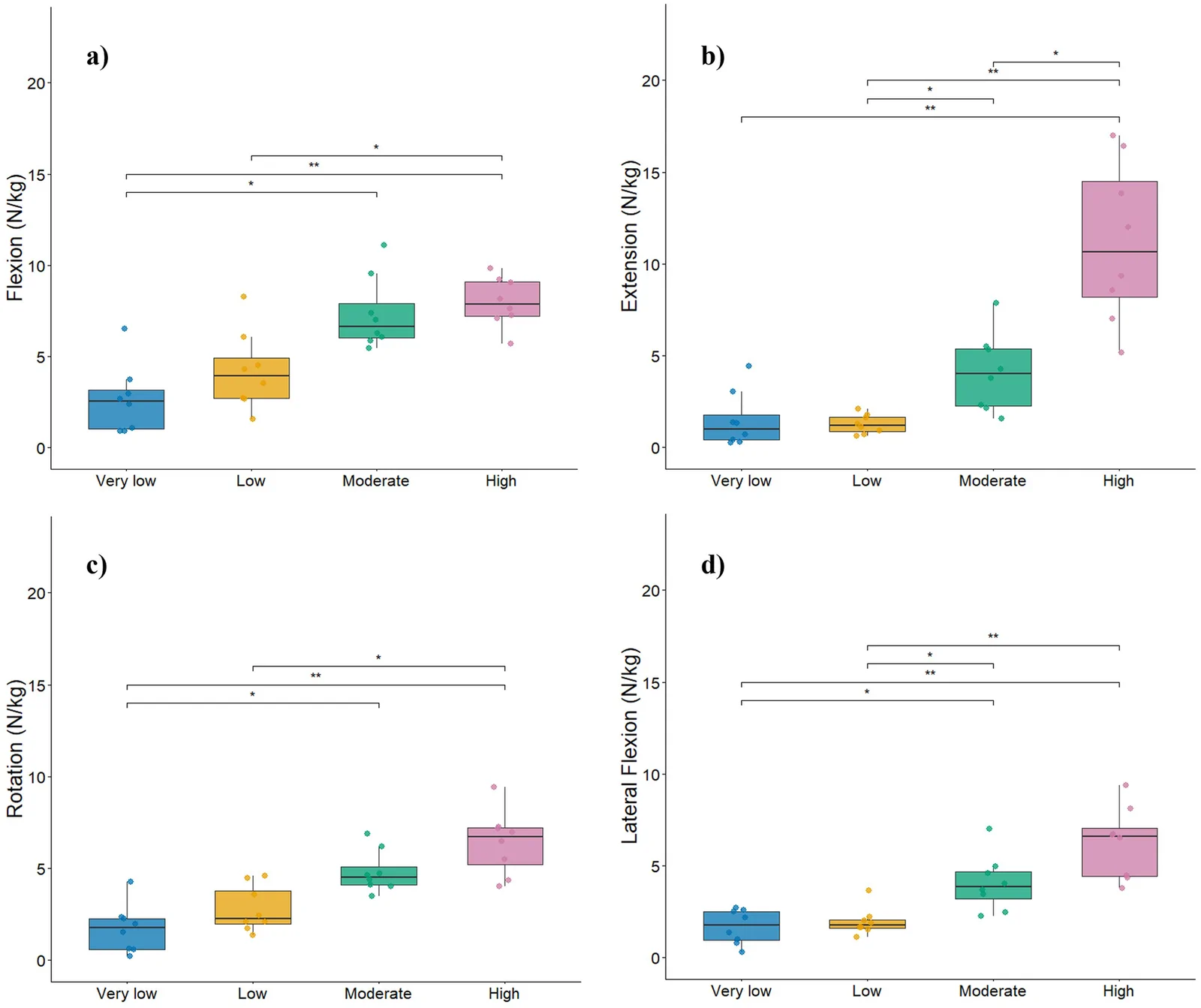
The relationship between MMT Trunk-Hip and maximal isometric trunk strength relative to body weight (N/kg) of flexion **(a)**, extension **(b)**, rotation **(c)**, and lateral flexion **(d)** para-athletes were grouped based on MMT Trunk-Hip scores: Very low (blue), Low (yellow), Moderate (green), and High (pink). Boxplots represent the median and interquartile range (IQR). Statistical significance from pairwise *post hoc* tests is indicated as * *p* < 0.05, ** *p* < 0.01, and *** *p* < 0.001.

## 4 Discussion

The aim of this study was to examine isometric trunk strength in seated para-athletes and compare it with 1) MMT Trunk-Hip scores, and 2) isometric trunk strength in a reference group without physical impairments. The main findings were that higher MMT Trunk–Hip scores were associated with higher isometric trunk strength, and that Para-athletes in the High group demonstrated strength levels not significantly different from those in the Ref AB. In contrast, the Very Low and Low groups showed consistently lower strength across all movement directions. The Moderate group demonstrated a more heterogeneous pattern, with strength levels varying with movement directions. In flexion and rotation, strength was higher than in the Very Low group and not significantly different to the High and Ref AB groups, whereas in extension and lateral flexion, strength was intermediate between the lower groups and the High and Ref AB groups.

The overall association between MMT Trunk–Hip scores and isometric trunk strength supports the notion that clinical assessment of trunk and hip muscle strength captures meaningful differences in the ability to generate trunk force. This is consistent with previous findings demonstrating associations between MMT and quantitative strength measures in the upper and lower extremities (33). The relationship appeared particularly evident at the lower and higher ends of the functional spectrum, as reflected by the clear differences between the Very Low and High groups. Previous research in individuals with SCI indicates that trunk strength is influenced by the severity and level of neurological impairment. Wheelchair users with complete injuries (AIS A–B) have been shown to exhibit lower trunk strength than ambulatory individuals with incomplete injuries (AIS C–D), particularly in extension (34). Furthermore, individuals with SCI at levels Th7 to L3 (AIS A–B) demonstrate considerable variability in trunk strength (28), suggesting that both injury severity and neurological level contribute to the capacity to generate trunk force.

Previous research in para-athletes and individuals with physical impairments further supports the association between impairment and isometric trunk strength. Individuals with amputation have been shown to exhibit greater trunk extension strength than individuals with paraplegia and strength levels not significantly different to a reference group without impairment (29). Similarly, wheelchair racers with clinically unimpaired trunk function have demonstrated greater isometric trunk flexion strength than those with impaired trunk function (7). In wheelchair rugby and wheelchair basketball, athletes with greater trunk impairment have also been shown to exhibit lower trunk flexion strength (30), while higher wheelchair basketball sport classes have been associated with greater strength in flexion, extension, and lateral flexion (27). Together, these findings support the present results and suggest that clinical assessments of trunk and hip muscle strength can distinguish meaningful differences in isometric trunk strength.

Although the overall findings support a relationship between MMT scores and isometric trunk strength, this relationship was not uniform across movement directions. This was particularly evident in the Moderate group, which demonstrated strength levels not significantly different from the High group in flexion and rotation but intermediate strength in extension and lateral flexion. One possible explanation is the heterogeneity of the underlying neurological conditions represented within this group. In SCI, variation in neurological level and injury completeness (e.g., AIS A–D) can result in substantial differences in preserved motor function (35), and individuals with spina bifida may also present variation in motor function (36). Thus, individuals with similar MMT Trunk–Hip scores may nevertheless have different patterns of preserved muscle function and different capacities to generate force in specific movement directions. Direction-specific differences in trunk function are also reflected in para-sport classification systems. For example, wheelchair basketball classification distinguishes athletes partly according to their ability to perform different trunk movements, with higher sport classes generally demonstrating greater capacity for flexion, rotation, and lateral flexion (37).

The direction-specific pattern observed in the Moderate group may also be related to differences in muscle innervation and recruitment, as well as the use of compensatory movement strategies. Trunk rotation involves the coordinated contribution of multiple muscles, including the internal and external obliques, erector spinae, quadratus lumborum, iliacus, and psoas, whereas the rectus abdominis primarily contributes to stabilization (38). Because these muscles receive innervation from different spinal segments, the effect of a neurological lesion may vary according to its level and completeness. In addition, trunk movements can be supported by muscles outside the trunk. Individuals with reduced trunk muscle strength following SCI have been shown to use altered muscle recruitment patterns, including increased activation of the pectoralis major, latissimus dorsi, and trapezius (39). Such recruitment may facilitate force transmission between the pelvis, shoulder girdle, and upper trunk and thereby contribute to the generation of rotational force even when intrinsic trunk muscle function is impaired. Previous research has similarly suggested that compensatory strategies involving the upper body may contribute to the preservation of trunk rotational strength following SCI (40, 41). These mechanisms may partly explain why rotational strength in the Moderate group was relatively preserved compared with strength in other movement directions. However, because these mechanisms were not directly assessed in the current study, this interpretation remains speculative.

The difference between flexion and extension strength provides another example of the direction-specific nature of trunk strength. In the High and Ref AB groups, extension strength was greater than flexion strength, consistent with previous findings in seated position with participants without impairment (42). In contrast, flexion strength appeared to be greater than extension strength in the Low and Moderate groups. One possible explanation may be differences in the muscles contributing to seated trunk movement and their respective patterns of innervation (43). Hip flexion is primarily generated by muscles such as the iliopsoas, which receive innervation from the upper lumbar segments (L1–L4), whereas hip extension relies more heavily on muscles such as the gluteus maximus, which receives innervation from the lower lumbar and sacral segments (L4–S2). Neuromusculoskeletal impairments affecting the lower lumbar or sacral segments, as may occur in conditions such as SCI or spina bifida, could therefore potentially have a greater effect on the muscles contributing to seated trunk extension than on those contributing to flexion. However, this explanation should be interpreted cautiously, as the current study did not directly assess muscle activation or neurological level in relation to the observed strength differences. The relationship between flexion and extension strength in athletes with spinal conditions therefore warrants further investigation in larger and more homogeneous populations.

The findings also have implications for how trunk strength impairment is assessed in the context of para-sport classification. The association between MMT scores and isometric strength indicates that MMT captures important differences in force-generating capacity. However, MMT should not necessarily be interpreted as a direct measure of maximal strength. MMT is based on qualitative clinical judgement and has ceiling effects, limiting its ability to distinguish between higher levels of muscle strength (44). Furthermore, MMT assesses muscle strength in relatively isolated muscle actions rather than the capacity to generate force during functional or sport-specific tasks. Quantitative strength measures may therefore provide complementary information. At the same time, isolated strength measures have been shown to relate only weakly to sport performance (45), and isometric strength may not be resistant to training. When neural innervation is intact and an individual is able to increase voluntary muscle activation, contractile strength can increase with training (46). Given that isometric strength is a trainable capacity (1, 22), its use as a sole indicator of impairment for classification may therefore be problematic, as previously argued by Beckman and colleagues (21).

Taken together, these considerations suggest that MMT and isometric strength testing should not necessarily be viewed as competing approaches, but rather as measures capturing different aspects of strength. MMT may provide information about the presence and extent of muscle strength impairment, whereas quantitative isometric testing provides information about the athlete's force-generating capacity and may reveal differences between movement directions that are not fully captured by MMT. In classification, where the assessment of impairment should be resistant to training and relevant to sport-specific activity, neither measure is likely to provide a complete representation of the athlete's functional capacity when considered in isolation.

This is consistent with the broader principle of sport-specific classification outlined in the IPC Classification Code (25), where athletes are assessed in relation to the activities required in their sport and, where relevant, using their adaptive equipment. In para-canoe classification, for example, MMT-based assessments of trunk and hip strength are combined with sport-specific testing performed in the athlete's adaptive equipment, including on-water evaluations (20, 26). Results from the clinical assessment and sport-specific testing are then considered together in the final class allocation. The present findings support such a multidimensional approach by demonstrating that clinical MMT scores are related to quantitative trunk strength, while also showing that this relationship varies between movement directions and levels of impairment.

### 4.1 Methodological considerations and limitations

Isometric trunk strength in seated para-athletes has previously been assessed using different load-cell setups, including handheld dynamometers, cable-mounted systems, and strength rigs (16, 27, 47). In the current study, a strength rig was used to provide a stable and standardized testing setup. However, the rig measured linear force rather than torque (Nm). Consequently, the results were not normalized to the individual's trunk dimensions or lever arm. Future studies should consider measuring the distance between the force transducer and the relevant anatomical landmark to allow calculation of torque. At present, however, there is limited standardization regarding how trunk strength should be expressed and which lever arm should be used for normalization. Further methodological work is therefore needed to establish standardized procedures for measuring seated isometric trunk strength.

Rotational strength is particularly challenging to assess because force generation involves the interaction between the pelvis, trunk, and upper body. In the current study, compensatory strategies were observed, particularly among para-athletes with lower MMT Trunk–Hip scores. These included contributions from trunk flexion and head or upper-body positioning. Although such strategies may reflect the functional capacity of para-athletes to generate rotational force, they also reduce the extent to which the measured force can be interpreted as isolated trunk muscle strength. Future studies should therefore consider standardized monitoring or quantification of compensatory movement strategies during rotational testing.

The MMT Trunk–Hip scores used in the current study reflect motor impairment. Sensory impairment was not assessed separately. In participants with SCI or spina bifida, this limits the possibility of relating strength capacity to the full neurological impairment profile. Furthermore, the study population was heterogeneous, although predominantly consisting of athletes with spinal conditions. Only three participants had lower-limb amputation, limiting the generalizability of the findings to athletes with amputation or other impairment types.

The grouping of participants according to MMT Trunk–Hip scores was also a methodological consideration. Cluster analysis did not identify a clear optimal number of groups, as silhouette analysis did not support a distinct cluster structure. The four-group solution was therefore based on a pragmatic balance between maintaining meaningful differences in MMT scores between groups and retaining sufficient participants within each group. The grouping strategy was inspired by Tweedy & Vanlandewijck (44) when no natural classes occur. Although three-, four-, and five-group solutions were examined, the final boundaries were not derived from established classification criteria and were not intended to inform sport class allocation. The differences observed between groups should therefore be interpreted as exploratory.

The standardized testing position, with the hips and knees at 90° and the pelvis and legs strapped, was made as recommended in the population without physical impairment (48). However, Para-athletes typically optimize their seating position, including seat tilt and backrest height, according to their sport and individual needs. The present findings may therefore not fully represent trunk strength in sport-specific seating positions. In addition, the degree of fixation may have varied between participants because of differences in body morphology and soft tissue around the pelvis and legs.

Sport-specific training may also have influenced trunk strength capacity. As the participants represented seated para-athletes from different sports, differences in training demands may have contributed to between-athlete variation in strength. The sample included only male para-athletes, which limits generalizability to female athletes. Future studies should therefore include both sexes and a broader range of sports and impairment types.

Finally, the cross-sectional design limits conclusions regarding the trainability of isometric trunk strength. Although the findings support the use of MMT and isometric strength testing as complementary measures, longitudinal studies are needed to determine how these measures respond to training and whether changes in isometric strength can be distinguished from changes in underlying impairment.

## 5 Conclusion

In seated para-athletes with neuromusculoskeletal impairments affecting the trunk and/or legs, higher MMT Trunk–Hip scores were associated with higher isometric trunk strength. However, the relationship varied between movement directions, indicating that MMT and isometric strength testing provide complementary information about trunk strength. MMT may provide an indication of the presence and extent of muscle strength impairment, whereas quantitative isometric strength testing provides information about force-generating capacity and direction-specific strength differences. Combining these assessments may therefore be valuable for performance enhancement by providing a more comprehensive understanding of an athlete's strength profile and helping to identify and monitor trainable strength capacity. In the context of para-sport classification, the findings support the use of MMT as one component of a multidimensional assessment approach, while quantitative isometric strength measures may provide complementary information but should be interpreted with consideration of their trainability.

## Data Availability

The original contributions presented in the study are included in the article/Supplementary Material, further inquiries can be directed to the corresponding author.

## References

[1] Akuthota V Nadler SF. Core strengthening. Arch Phys Med Rehabil. (2004) 85:86–92. 10.1053/j.apmr.2003.12.005 15034861

[2] Ershad N Kahrizi S Abadi MF Zadeh SF. Evaluation of trunk muscle activity in chronic low back pain patients and healthy individuals during holding loads. J Back Musculoskelet Rehabil. (2009) 22(3):165–72. 10.3233/BMR-2009-0230 20023346

[3] Enoka RM. Neuromechanics of Human Movement. Champaign, IL: Human Kinetics (2008).

[4] Kibler WB Press J Sciascia A. The role of core stability in athletic function. Sports Med. (2006) 36(3):189–98. 10.2165/00007256-200636030-00001 16526831

[5] International Paralympic Committee (n.d.a) Classification: Available online at: https://www.paralympic.org/classification (Accessed April 2, 2026)

[6] International Paralympic Committee (n.d.b) Sports: Available online at: https://www.paralympic.org/sports (Accessed April 2, 2026)

[7] Vanlandewijck YC Verellen J Tweedy S. Towards evidence-based classification in wheelchair sports: impact of seating position on wheelchair acceleration. J Sports Sci. (2011) 29(10):1089–96. 10.1080/02640414.2011.576694 21756128

[8] Curtis KA Kindlin CM Reich KM White DE. Functional reach in wheelchair users: the effects of trunk and lower extremity stabilization. Arch Phys Med Rehabil. (1995) 76(4):360–7. 10.1016/S0003-9993(95)80662-8 7717837

[9] Haydon DS Millett EL. Wheelchair racing biomechanics and performance considerations. In: Griggs K Arnet U van der Slikke R Flueck JL, editors. The Routledge handbook of disability sport science. 1st ed. London: Routledge; 2026. p. 176–90.

[10] Hyde A Hogarth L Sayers M Beckman E Connick MJ Tweedy S. The impact of an assistive pole, seat configuration, and strength in paralympic seated throwing. Int J Sports Physiol Perform. (2017) 12(7):977–83. 10.1123/ijspp.2016-0340 27967276

[11] Mason BS Altmann VC Goosey-Tolfrey VL. Understanding the impact of trunk and arm impairments on wheelchair rugby performance during competition. Int J Sports Physiol Perform. (2019) 14(5):612–9. 10.1123/ijspp.2018-0204 30427241

[12] van der Slikke RMA Rietveld T Goosey-Tolfrey VL Berger M. Trunk function: the core of mobility performance in wheelchair tennis. Front Sports Act Living. (2026) 8:1783088. 10.3389/fspor.2026.1783088 41958819 PMC13057481

[13] Wileman TM McKay MJ Hackett DA Watson TJ Fleeton J Fornusek C. Guiding evidence-based classification in para sporting populations: a systematic review of impairment measures and activity limitations. Sports Med. (2025) 55(2):341–91. 10.1007/s40279-024-02132-y 39572519 PMC11946994

[14] Mann DL Tweedy SM Jackson RC Vanlandewijck YC. Classifying the evidence for evidence-based classification in paralympic sport. J Sports Sci. (2021) 39(sup1):1–6. 10.1080/02640414.2021.1955523 34369294

[15] Tweedy SM Beckman EM Connick MJ. Paralympic classification: conceptual basis, current methods, and research update. PM&R. (2014) 6(8 Suppl):S11–S17. 10.1016/j.pmrj.2014.04.013 25134747

[16] O'Connor SR Fagher K Williamson S Pluim BM Ardern CL Van Rensburg DCJ. Assessment of muscle strength in para-athletes: a systematic review of observational studies. Sports Med Health Sci. (2022) 4(4):225–38. 10.1016/j.smhs.2022.07.004 PMC9806714 36600967

[17] Tweedy SM Williams G Bourke J. Selecting and modifying methods of manual muscle testing for classification in paralympic sport. Eur J Adapt Phys Act. (2010) 3(2):7–16. 10.5507/euj.2010.005

[18] Bohannon RW. Reliability of manual muscle testing: a systematic review. Isokinet Exerc Sci. (2018) 26(4):245–52. 10.3233/IES-182178

[19] Cuthbert SC Goodheart GJ. On the reliability and validity of manual muscle testing: a literature review. Chiropr Osteopat. (2007) 15(1):4. 10.1186/1746-1340-15-4 17341308 PMC1847521

[20] Rosén J. Performance Determinants and Classification in Paracanoe. Stockholm: Swedish School of Sport and Health Sciences (2021).

[21] Beckman EM Connick MJ Tweedy SM. Assessing muscle strength for the purpose of classification in paralympic sport: a review and recommendations. J Sci Med Sport. (2017) 20(4):391–6. 10.1016/j.jsams.2016.08.010 27692576

[22] Oranchuk DJ Storey AG Nelson AR Cronin JB. Isometric training and long-term adaptations: effects of muscle length, intensity, and intent. Scand J Med Sci Sports. (2019) 29(4):484–503. 10.1111/sms.13375 30580468

[23] Gebeyehu TF Mong ER Thalheimer S Vaccaro AR Harrop J. Epidemiology of spinal cord injury and associated mortality, past and present. World Neurosurg. (2024) 192:e494–505. 10.1016/j.wneu.2024.10.009 39414135

[24] Schwartz S Cohen M Herbison G Shah A. Relationship between two measures of upper extremity strength: manual muscle test compared to hand-held myometry. Arch Phys Med Rehabil. (1992) 73:1063–8. 1444773

[25] International Paralympic Committee (2025) IPC Classification code: Available online at: https://www.paralympic.org/sites/default/files/2025-02/IPC%20Classification%20Code%2001_01_2025.pdf (Accessed April 24, 2026).

[26] International Canoe Federation (2018) *ICF Paracanoe Kayak Classification Manual*: Available online at: https://www.canoeicf.com/sites/default/files/icf_paracanoe-kayak_classification_manual_19-08-2018.pdf (Accessed April 2, 2026).

[27] Schaaf K Nquiti M Lassner P Bersiner K Gehlert S Abel T. Field-based evaluation of trunk strength using a novel in-chair assessment system in elite wheelchair athletes. Sci Rep. (2026) 16:8967. 10.1038/s41598-026-44257-2 41820639 PMC12988200

[28] Turoglu S Koç SS Shehu SU Yilmaz AE Sezer N Akınoğlu B. Investigating the relationship between spinal alignment and trunk muscle strength, sitting balance and function in spinal cord injury. J Spinal Cord Med. (2026) 49(5):962–73. 10.1080/10790268.2025.2557079 41032383 PMC13591977

[29] Fortes JPA Hotta GH Aguiar DP Oliveira VBSD Oliveira FCD MATTOS B. Reliability of the isometric dynamometer in control, paraplegic, and amputee individuals. Acta Ortop Bras. (2023) 31:e255829. 10.1590/1413-785220233101e255829 36844127 PMC9949314

[30] Altmann VC Groen BE Groenen KH Vanlandewijck YC Van Limbeek J Keijsers NL. Construct validity of the trunk impairment classification system in relation to objective measures of trunk impairment. Arch Phys Med Rehabil. (2016) 97(3):437–44. 10.1016/j.apmr.2015.10.096 26551229

[31] Hislop H Montgomery J. *Daniels and Worthingham's muscle testing: techniques of manual examination*. 7th ed. Philadelphia: W.B. Saunders (2002).

[32] Field A. Discovering Statistics using IBM SPSS Statistics. 4th ed London: SAGE (2013).

[33] Aitkens S Lord J Bernauer E Fowler WM Jr Lieberman JS Berck P. Relationship of manual muscle testing to objective strength measurements. Muscle Nerve. (1989) 12(3):173–7. 10.1002/mus.880120302 2725546

[34] Gabison S Verrier MC Nadeau S Gagnon DH Roy A Flett HM. Trunk strength and function using the multidirectional reach distance in individuals with non-traumatic spinal cord injury. J Spinal Cord Med. (2014) 37(5):537–47. 10.1179/2045772314Y.0000000246 25229736 PMC4166188

[35] Stone WJ Stevens SL Fuller DK Caputo JL. Strength and step activity after eccentric resistance training in those with incomplete spinal cord injuries. Top Spinal Cord Inj Rehabil. (2018) 24(4):343–52. 10.1310/sci17-00052 30459497 PMC6241222

[36] Bendt M Seiger Å Hagman G Hultling C Franzen E Forslund EB. Adults with spina bifida: ambulatory performance and cognitive capacity in relation to muscle function. Spinal Cord. (2022) 60(2):122–8. 10.1038/s41393-021-00658-w 34262127

[37] International Wheelchair Basketball Federation. Player classification manual. IWBF. (2022). Available online at: https://cdn.prod.website-files.com/66a7ad387e177bc7c659bb87/67950b8e8a8182399d31d0c5_2021-IWBF-Classification-Manual-Version-202212-12%20small.pdf (Accessed Sept 21, 2026).

[38] Andersson EA Grundström H Thorstensson A. Diverging intramuscular activity patterns in back and abdominal muscles during trunk rotation. Spine. (2002) 27(6):E152–60. 10.1097/00007632-200203150-00014 11884920

[39] Bjerkefors A Carpenter MG Cresswell AG Thorstensson A. Trunk muscle activation in a person with clinically complete thoracic spinal cord injury. J Rehabil Med. (2009) 41(5):390–2. 10.2340/16501977-0336 19363574

[40] Seelen HAM Potten YJM Drukker J Reulen JPH Pons C. Development of new muscle synergies in postural control in spinal cord injured subjects. J Electromyogr Kinesiol. (1998) 8(1):23–34. 10.1016/S1050-6411(97)00002-3 9667031

[41] Milosevic M Yokoyama H Grangeon M Masani K Popovic MR Nakazawa K. Muscle synergies reveal impaired trunk muscle coordination strategies in individuals with thoracic spinal cord injury. J Electromyogr Kinesiol. (2017) 36:40–8. 10.1016/j.jelekin.2017.06.007 28734171

[42] Kocjan A Sarabon N. Assessment of isometric trunk strength - the relevance of body position and relationship between planes of movement. J. Sports Sci. Med. (2014) 13:365–70. 24790491 PMC3990891

[43] Platzer W. Color Atlas of Human anatomy. Vol. 1, Locomotor System. 7th ed Stuttgart: Thieme (2015).

[44] Tweedy SM Vanlandewijck YC. International paralympic committee position stand—background and scientific principles of classification in paralympic sport. Br J Sports Med. (2011) 45(4):259–69. 10.1136/bjsm.2009.065060 19850575

[45] Reiman MP Manske RC. The assessment of function: how is it measured? A clinical perspective. J Man Manip Ther. (2011) 19(2):91–9. 10.1179/106698111X12973307659546 22547919 PMC3172944

[46] Jayaraman A Thompson CK Rymer WZ Hornby TG. Short-term maximal-intensity resistance training increases volitional function and strength in chronic incomplete spinal cord injury: a pilot study. J Neurol Phys Ther. (2013) 37(3):112–7. 10.1097/NPT.0b013e31828390a1 23673372

[47] Da Silva Santos S Krishnan C Alonso AC Greve JMDA. Trunk function correlates positively with wheelchair basketball player classification. Am J Phys Med Rehabil. (2017) 96(2):101–8. 10.1097/PHM.0000000000000548 27323325

[48] Stark T Walker B Phillips JK Fejer R Beck R. Hand-held dynamometry correlation with the gold standard isokinetic dynamometry: a systematic review. PM&R. (2011) 3(5):472–9. 10.1016/j.pmrj.2010.10.025 21570036

